# Sulphurous Acid as a Bleach

**Published:** 1881-10

**Authors:** 


### SULPHUROUS ACID AS A BLEACH.

For bleaching wool, silk, and straw, sulphurous acid, or sulphurous anhydride, as it is frequently called, has long been employed, and the old method of generating it for the purpose by burning sulphur is still the most common. But the operation is attended with more or less uncertainty, and the want of uniformity in the results is frequently a source of annoyance to the bleacher, if not of positive loss. Now, this uncertainty is mainly due to the varying conditions of the atmosphere of the bleach chamber. The temperature to which the textiles are subjected there is far from being uniform. The proportion of sulphurous acid in one portion differs widely from that in another, and the acid is far from pure.

The impurities in the gas come from the impure form of commercial sulphur, which must of necessity be used. The rate and direction of the circulation of the gas must depend upon the rate of the combustion ; which, at first sluggish, becomes active as the sulphur melts, until the liquid reaches so high a temperature that a portion of it is volatilized unconsumed and rises in the form of vapor, mingled with the sulphurous acid. The latter, familiarly known as a fire extinguisher, prevents the combustion of the volatilized sulphur until perchance it reaches the fabric to be bleached, where meeting with conditions favorable to combustion, it is consumed, producing a slight stain. This is especially noticeable on silk but moderately dampened.

Bleaching by means of burning sulphur must indeed be regarded as a rude process, nor can it be called econominal, for, in order to guard against the effects of its uncertainty, a large quantity of sulphur is employed, the length of time during which the goods are suspended in the bleach chamber is prolonged, and the number of times they are so suspended is multiplied. Twenty-four pounds of sulphur are roughly estimated as necessary to bleach two thousand yards of woolen fabric during an exposure lasting twelve hours, and in practice it is washing with soda lye and fair water intervening, subjected to two and sometimes to three such exposures before the bleaching is regarded as finished. Sulphurous acid is very soluble in water, and vats containing solutions of it have been substituted for the chamber ; but these solutions soon undergo a change, a portion of the sulphurous acid being converted into sulphuric, which impairs the softness of the fiber, if not its strength.

The cheapness with which the soluble salts of sulphurous acid can be made has led to attempts at their introduction ; and the facility with which they may be decomposed, eliminating pure sulphurous acid, is also in their favor. Further and careful experiments on their employment would seem to be demanded by the wants of the bleacher.

Sulphurous acid is readily condensed into a liquid, being at ordinary temperatures liquified by a pressure of two atmospheres ; and its preparation in the manufacturing laboratory, and its sale in suitable condensers, were proposed years ago, but its use did not at that time receive the sanction of practical men. Since then our means of condensing gases have been greatly improved, and Prof. Raoulpet Pictet, of the University of Geneva, Switzerland, than whom no better authority can be desired, strongly recommends the condensed gas for bleaching purposes. Recently he condensed into a strong vessel of the capacity of 1 liter, 225 liters of the gas, which, probably from its purity, had, when allowed to escape into the atmosphere of the chamber, great penetrative power, passing rapidly through fabrics almost impermeable by air.

Bleached in this manner, the most delicate silk fiber loses none of its elasticity or strength. A number of Swiss silk manufacturers have already adopted the use of the condensed gas, which promises soon to become a commercial article in all civilized silk-producing countries, and ere long to render the practice of bleaching wool and woolens by gas evolved from burning sulphur in the bleachery a thing of the past.—*Textile Record.*

